# Synergistic Effect of *Bacillus* and Chitosan: From Flocculation to Enhanced Antimicrobial Activity

**DOI:** 10.3390/antibiotics14040412

**Published:** 2025-04-17

**Authors:** Selena Dmitrović, Nataša Lukić, Ivana Danilov, Vanja Vlajkov, Jovana Grahovac, Aleksandar Jokić

**Affiliations:** Faculty of Technology Novi Sad, University of Novi Sad, Bulevar cara Lazara 1, 21000 Novi Sad, Serbia; selena.dmitrovic@uns.ac.rs (S.D.); nlukic@tf.uns.ac.rs (N.L.); ivana.pajcin@uns.ac.rs (I.D.); johana@uns.ac.rs (J.G.); jokic@uns.ac.rs (A.J.)

**Keywords:** *Bacillus* sp., flocculation, fruit juice, food industry wastewaters, downstream processing, biomass recovery

## Abstract

Eco-friendly pest management solutions are acknowledged as a crucial element in shaping the future of agriculture through sustainable practices. Achieving the maximum viable cell concentration while being cost-effective is the main goal of the downstream processing for efficient biomass-based microbial biopesticide production. The purpose of this study was to determine the effectiveness of chitosan flocculation in recovering bacterial *Bacillus* sp. BioSol021 biomass from broth cultivated using fruit juice industrial effluent as a medium, with the hypothesis of the synergistic effect of microbial and biopolymer components in phytopathogen suppression. Second-order polynomial models were used to calculate the influence of chitosan concentration and mixing speed on flocculation efficiency, settling velocity, and antibacterial activity against *Aspergillus flavus* (i.e., the inhibition zone diameter). The response surface approach, followed by desirability function optimization and the genetic algorithm were applied. The optimal values achieved in this study were 97.18%, 0.0369 mm/s, and 74.00 mm for flocculation efficiency, settling velocity, and inhibition zone diameter, respectively. The obtained results suggest that chitosan can be used as a flocculation agent for effective downstream processing, but also has a positive effect on the final product antimicrobial activity.

## 1. Introduction

From the perspective of sustainable agriculture evolution, the development of eco-friendly solutions in pest management emerges as one of the most significant challenges. Biopesticides including microbial cells as active components are one of the most promising alternatives to chemical choices, emphasizing the members of the *Bacillus* genus as powerful antimicrobial agents with remarkable capabilities for synthesizing a wide range of beneficial components. Their plant growth-promoting (PGP) attributes, including phosphate solubilization, nitrogen fixation, indole-3-acetic acid (IAA) and hydrogen cyanide (HCN) production, siderophores, hydrolytic enzymes, and antimicrobial compound synthesis, have been reported as key contributors to the multiple mechanisms of *Bacillus* representatives’ biocontrol activity [1]. Additional efforts are being made to make the biotechnological production of microbial biopesticides more efficient, considering economic and ecological aspects, making them more competitive on the global market of pesticides [2]. Utilization of industrial effluents is a possible valorization route to generate products with added value, and food industry wastewater is a particularly interesting option as a possible raw material for microbial biopesticide synthesis [3]. About 75% of organic compounds in wastewater generated by the food industry consist of fats, carbohydrates, proteins, volatiles, and amino acids, while inorganic compounds include sodium, potassium, chlorine, sulfur, calcium, phosphate, ammonium salts, magnesium, bicarbonate, and heavy metals. Nitrogen and phosphorus are key components, and studies have focused on their removal [4]. Fruit juice wastewater (FJW) contains a significant load of organic contaminants (organic acids and sugars) and suspended solid fruit remains, thus exhibiting a high chemical oxygen demand (COD), low pH value, and low concentrations of phosphorus and nitrogen [5,6]. Fruit juice production generates significant volumes of wastewater (approximately 10 L/1 L of fruit juice) following the cascade of operations including soaking, washing, blanching, extraction, and packing [5,7]. Based on its composition and the generated volumes, FJW requires proper treatment to align with the legislative requirements prior to its discharge into natural recipients [6]. However, FJW is highly biodegradable and contains fewer non-degradable compounds compared to wastewater from other industrial sectors [8]. The utilization of fruit juice waste in biotechnological production aligns with the principles of a circular economy, based on the effluents’ valorization in terms of material reuse and putting them back into the supply chain, providing economic benefits from environmental losses. The European Environment Agency (EEA) emphasized the importance of switching from a linear to circular economy concept and defined it as a crucial milestone to attain by 2050, through developing and implementing novel solutions based on waste utilization [9]. Taking into account the expected growth of the fruit juice industry and value, estimated at USD 216.6 billion by 2032, the future of this industry is highly dependent on achieving sustainability goals and achieving social development and environmental preservation [7].

The downstream treatment of microbial biopesticides based on bacterial biomass is reliant on achieving the highest concentration of viable cells while being cost-effective to ensure market competitiveness [9,10]. A relatively simple physico-chemical harvesting method is coagulation–flocculation. This method begins with the coagulation process, which includes the use of positively charged compounds to destabilize colloidal particles in suspension, followed by a second stage (flocculation), which agglomerates destabilized particles into larger ones, leading to deposition owing to their weight [11,12]. The coagulation–flocculation process often includes the use of inorganic metal salts such as ferric chloride, ferrous sulfate, ferric chloro-sulfate, and aluminum sulfate. Polymerized metal coagulants (e.g., poly-aluminum chloride) are increasingly used for water treatment due to their lower cost and greater availability [11]. Over time, these chemical coagulants exhibit a harmful effect on human health, leading to the development of many diseases, including Alzheimer’s disease and cancer. The limitations of mineral-based coagulants have led to the quest for non-toxic, biodegradable organic coagulants that are more ecologically friendly in both manufacturing and usage [12].

A biopolymer coagulant such as chitosan is a suitable replacement for metal coagulants, especially in light of the recent global uprise in the popularity of green technology. In addition, chitosan is safe, natural, and eco-friendly [13]. Chitin and chitosan are produced from a variety of organisms, including mollusks, crustaceans, arthropods, fungus, scales, and algae. Chitosan has been used to coagulate and flocculate wastewater from a variety of industries, including food processing, mining, brewing, pharmaceuticals, and sewerage. Numerous factors, including pH, the effluent water’s characteristics, and the amount of coagulant used, can influence the coagulation–flocculation process. The optimized levels of the aforementioned factors have to be combined for the process to produce the greatest results. Therefore, it is advised to use a jar test to identify the ideal operating parameters for these factors [14]. The traditional chitosan-based coagulation–flocculation process method (changing one interaction parameter while maintaining the others unchanged) is unsustainable, as well as time- and energy-consuming. Optimal conditions can be achieved by identifying the non-linear dependencies and interactions between the independent variables using the response surface methodology (RSM). Consequently, the majority of the drawbacks of the conventional coagulation method are resolved [13]. Besides the aforementioned beneficial aspects of using chitosan as a flocculant, numerous studies based on in vitro analyses have addressed the antimicrobial activity of chitosan against bacterial and fungal plant pathogens. Besides representatives of the *Monilinia*, *Penicillium*, *Colletotrichum*, *Botrytis*, and *Sclerotinia* genera, the antifungal activity of chitosan was also detected against the *Aspergillus* species, and the mechanism of action was described through the fungal morphology alterations caused by plasma membrane permeabilization and the consequent intracellular material leakage [15,16]. An additional effect of chitosan against the toxigenic *Aspergillus* species was demonstrated as a result of aflatoxin production inhibition due to chitosan’s chelating ability on metals, including zinc, magnesium, iron, and molybdenum, and induction of grains’ defense mechanisms by phenolic compound production [15]. Previous studies indicated the synergistic effect of simultaneous application of chitosan and endophytic bacteria belonging to the *Bacillus* genus with proven beneficial activity in agriculture practice, considering their positive impact on seed germination, plant growth, and protection against pathogenic species. Chitin and chitosan are effective agents in plant disease management and serve as fungicides, fertilizers (soil conditioners), bactericides, nematicides, and even antiperspirants. These substances enhance or trigger natural defense mechanisms in plants, acting as plant growth stimulants, elicitors for secondary metabolite production, growth regulators, and stress mitigators. Endophytic bacteria positively influence plant growth by promoting mechanisms such as the synthesis of growth hormones (phytohormones) and enhancing nutrient availability to plants (biofertilizers). Together, the combination of chitin/chitosan with endophytic bacteria offers synergistic benefits, promoting plant growth and providing protection against biotic diseases [17]. Aflatoxigenic *Aspergillus* species present one of the major challenges for feed and food safety, considering the possible aflatoxin transfer from cereal (predominantly maize) fields across the entire food chain [18]. Considerations related to their both acute and chronic negative effects on animal and human health, depending on the exposure concentration related to environmental condition-triggered activation of aflatoxin synthesis metabolic pathways [19], explain the rationale for choosing the aflatoxigenic *Aspergillus flavus*, isolated from corn, as the test plant pathogen in this study.

According to the literature data, this study hypothesizes that chitosan application in the biotechnological production of microbial biopesticides plays an important role in the downstream process, and provides an additional beneficial effect regarding the overall antimicrobial activity as a result of a synergistic effect of microbial and biopolymer components. This study also addresses the question of suitability of the wastestream from juice production utilization in terms of designing a biotechnological solution within the concept of a circular economy. The main goal of this study was to investigate the effects of chitosan dosing and the flocculation mixing speed on the flocculation efficiency of *Bacillus* sp. BioSol021 cultivation broth obtained via the biological treatment of wastewater from the fruit juice industry to recover bacterial biomass for microbial biopesticide production. Additionally, the synergistic antimicrobial effect of chitosan and bacterial biomass against the aflatoxigenic fungus *Aspergillus flavus* was assessed. Finally, an evaluation of the flocculation factors’ effects on the settling velocity and flocculated biomass antimicrobial activity was conducted. RSM and GA were used to optimize the flocculation process variables (mixing speed and flocculant dosage) to analyze the trade-offs among the set downstream procedure optimization objectives.

## 2. Results

The flocculation experimental design (central composite, α value of 1.41421), including the varied independent variables and responses, is presented in Table 1, including nine different experimental combinations with two central point replications.

### 2.1. Flocculation Efficiency

Table 2 summarizes the results of variance analysis (ANOVA) for the second-degree polynomial model for flocculation efficiency. The polynomial model for flocculation efficiency was significant (*p*-value = 0.0124), which was further confirmed by the F-value of 13.8272 and the *p*-value for lack-of-fit (0.0978), which was not significant.

A signal-to-noise ratio of at least 4 is considered desirable, and adequate accuracy is an indicator of this ratio. The flocculation efficiency ratio of 10.5085 in this instance demonstrates that this model may be utilized to explore the design space and indicates an appropriate signal.

Table 3 provides the coefficients corresponding to the selected independent variables of the obtained regression equation for flocculation efficiency, including the coded values and actual variables values together with their associated *p*-values. The obtained findings show that the linear impact of the chitosan dose is statistically significant, whereas the other effects are not statistically significant.

**Figure 1:**
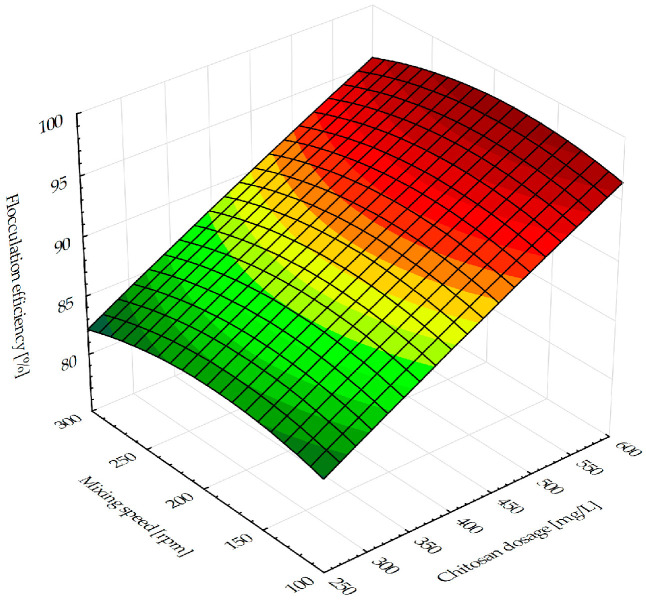
Influence of the chitosan dosage and rapid mixing speed on the flocculation efficiency.

Figure 1 displays a three dimensional response surface for the flocculation efficiency. Given the importance of the linear coefficient, it is recognized that the flocculation effectiveness in the experimental range rises linearly with increasing chitosan dosage.

### 2.2. Settling Velocity

The ANOVA for the quadratic mathematical model referring to settling velocity is shown in Table 4. The regression model was significant based on low *p*-values (<0.05), as confirmed by the model lack-of-fit *p*-value, which should be over 0.05. The value obtained in this study was 0.2827, showing the ability of the selected model to adequately fit the obtained experimental data.

About 9% of the variations in the flocculated broth settling velocity could not be explained by the quadratic model, according to the coefficient of determination. The ratio of 8.4735 for Adequate Precision suggests that the second-degree polynomial model for the response settling velocity is acceptable.

Table 5 shows the coefficients of the regression equation for the settling velocity, including the coded values and the real variable values, together with the related *p*-values. The results have revealed statistical significance for the linear (*b*_1_) and quadratic effects (*b*_11_) of chitosan dosage.

**Figure 2:**
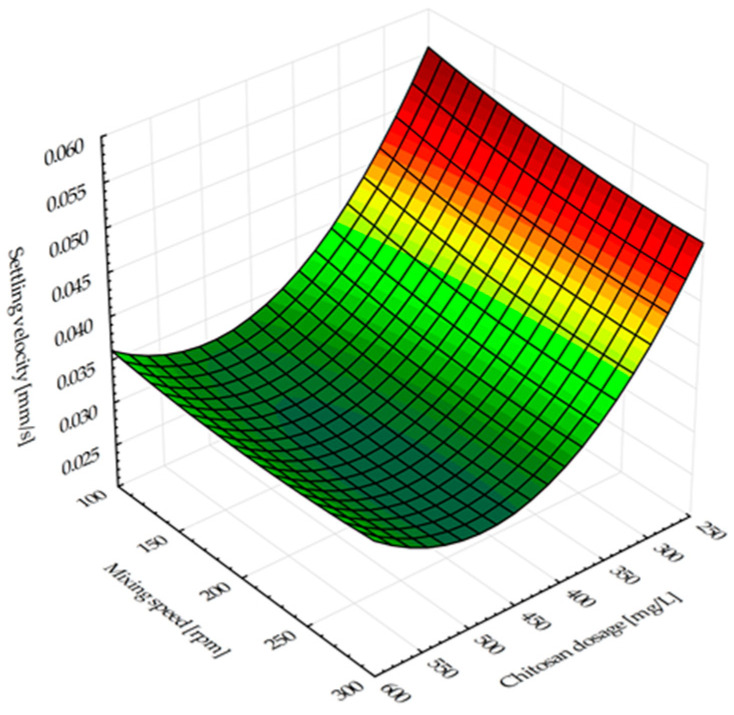
Influence of the chitosan dosage and rapid mixing speed on the settling velocity.

The effects of chitosan dosage and mixing speed as the independent variables on the settling velocity of the flocculated broth as the response are shown in Figure 2.

Figure 2 shows that, in the experimental range, the effects of the chitosan dosage exceed the influence of the mixing speed in terms of the settling velocity, and at greater agitation speeds, the reduction in the settling velocity is more evident.

### 2.3. Inhibition Zone Diameter

Table 6 shows the ANOVA research results for a quadratic model of the inhibition zone diameter.

The model’s *p*-value confirms the significance of the regression equation. The *p*-value for the inhibition zone diameter model’s lack-of-fit in this study, as indicated in Table 6, was 0.3067, indicating that the model was able to adequately fit the obtained experimental data. The relatively high R^2^ value (0.9643) implies that the model is suitable for adequate empirical data representation. The suitable precision ratio of 11.8916 suggests that the signal-to-noise ratio for the response related to inhibition zone diameter was adequate.

Table 7 provides the regression equation coefficients for the inhibition zone diameter, together with the corresponding *p*-values for the coded and real variable values. The obtained results showed that the quadratic effect of the mixing speed and the linear effects of both independent variables were statistically significant. The quadratic impact of the chitosan dosage was not significant. The interaction between the mixing speed and chitosan dosage in the regression equation was also statistically significant.

**Figure 3:**
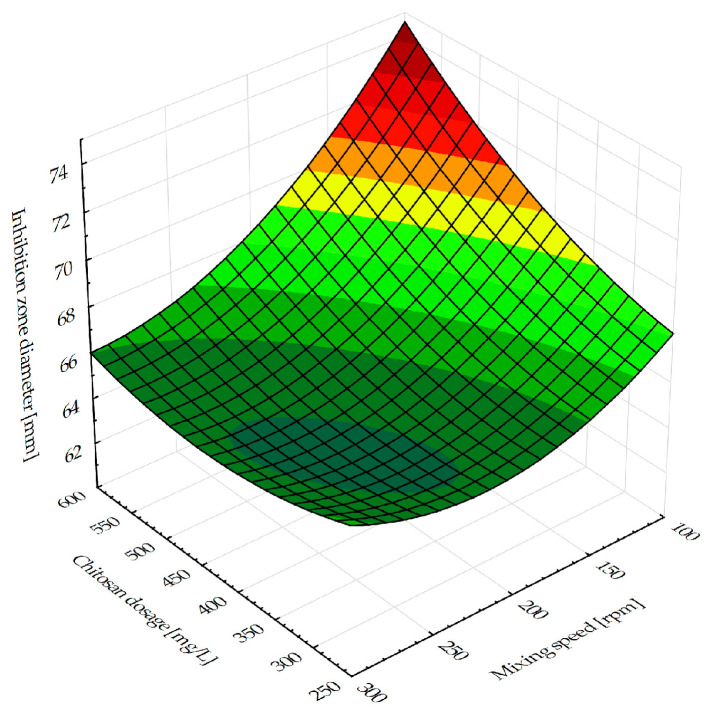
Influence of the chitosan dosage and rapid mixing speed on the inhibition zone diameter.

The antifungal activity against *Aspergillus flavus* was shown to be positively impacted by both the chitosan and cultivation broth. According to Figure 3′s findings on the impact of the mixing speed on the inhibition zone diameter, an increase in mixing speed causes an inhibition zone diameter reduction.

### 2.4. Optimization

**Figure 4:**
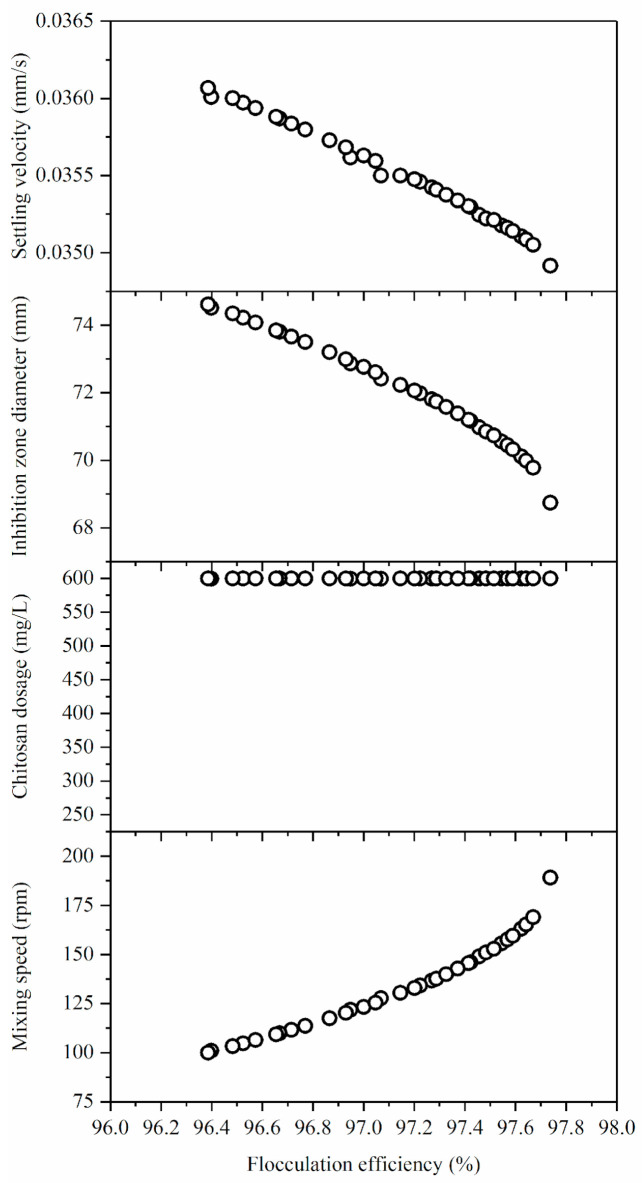
The decision space and Pareto front plot of the best possible solution set generated by the multi-objective genetic algorithm.

The desirability function (DF) and genetic algorithm (GA) were used to optimize the operating parameters during flocculation-based biomass harvesting of *Bacillus* sp. BioSol021 from the cultivation broth. Figure 4 shows the outcomes of the GA optimization.

Figure 4 shows the outcomes of the three-objective GA optimization. The coded NSGA-II in MATLAB software was used to solve the optimization issue and generate a set of Pareto front-optimal solutions. Two popular visualization techniques for the multi-dimensional Pareto front are scatter diagrams and parallel coordinates [20,21,22].

The Pareto front and the highest scoring prediction obtained by the DF method have coincided. The Pareto front results in plenty of optimal solutions, yet the choice of a compromise–optimal solution is difficult. While selecting an appropriate solution, the desired and previously defined optimization’s goal(s) can be taken into account. However, more trade-offs or compromises between the different objectives are necessary [9]. The Pareto front shows the contradictory relationship when considering flocculation efficiency, settling velocity, and inhibition zone diameter. In other words, when the chitosan dosage increases, the flocculation efficiency increases, but this is followed by a decrease in the settling velocity and inhibition zone diameter.

## 3. Discussion

The commercialization perspective of biopesticides, with an emphasis on microbial-based products, depends on the possibilities of providing sustainable and economically efficient solutions for their development. Regarding this question, scientists are joined in efforts to answer these challenges by creating advanced approaches addressing both the upstream and downstream stages of biotechnological production [23]. One of the promising solutions, aiming to improve the economic efficiency of the whole process, is the application of industrial effluents as a replacement for commercially used synthetic media for microbial growth. The other approach also investigated in the present study is defining the suitable downstream technology for the maximization of suppressive biocontrol activity of the final product, including the concentration of viable microbial cells and additional effect enhancement by introducing a biopolymer component [24]. Modelling of the observed bioprocess was performed using the dual approach, the response surface method (RSM) and machine learning methodology.

Statistical design application in experimental work enables systematic analysis and optimization of multiple parameters crucial for the observed system. Response surface methodology (RSM) is a mathematical approach which efficiently provides detailed information on both the independent and combined effects of all the independent variables. Relying on a limited number of planned experiments, RSM is a strategy for creating mathematical models based on experimental tests, resulting in the assessment of the impact of various variables, and providing answers regarding the optimal conditions for the desired outcomes. Previous studies [11,25,26], in addressing wastewater treatment, have documented the use of RSM as a suitable tool for coagulation–flocculation process optimization. Thus, the application of the RSM experimental design technique with this purpose was focused on the most important parameters of the coagulation–flocculation process, including the coagulant dosage and mixing speed. A true optimum is therefore achieved by observing the parameter interactions and the non-linear correlations between the investigated variables [27]. On the other hand, machine learning relies on a different approach, completing a task using computer-based learning from the provided datasets, without the necessity to provide detailed information on the investigated task and providing a broad range of possible applications, including classification, optimization, identification, and prediction. Following the growth of environmental consciousness, machine learning algorithms are increasingly used for wastewater treatment. The present study uses GA to investigate the optimal parameters for effective biomass flocculation [28], as an optimization technique that utilizes natural selection theory and biological evolution patterns, and a methodology of choice widely used in a broad range of research fields [29].

The flocculation process was observed using the graphical representation of the most relevant factors’ influence on the selected responses. The 3D-fitted response surface representing the influence of the chitosan dosage and the mixing speed on the flocculation efficiency is presented in Figure 1. The tendency of the flocculation efficiency increase following a chitosan dosage increase is exhibited at all mixing speeds within the investigated experimental range. The results obtained in the study addressing chitosan flocculation in microalgal–bacterial biomass harvesting [30] are consistent with those in this paper. The results represented in Figure 1 illustrate that at all mixing speeds, an efficiency greater than 90% was attained for chitosan concentrations over 450 mg/L. The addition of 672.5 mg/L of chitosan results in the highest flocculation efficiency of 98.83% (Table 1). At these chitosan dosage ranges, a plateau is reached and a further dosage increase (above 700 mg/L) could lead to slightly less efficient flocculation. Concerning the chitosan dose, the mixing speed appears to have less of an influence on the flocculation capabilities (Figure 1). The evident rise is visible for a rapid mixing speed interval reaching 200 rpm, while a further mixing speed increase results in a minor descent of flocculation efficiency. The described behavior is observed over the full range of chitosan dosage levels. At lower suspension agitation levels, the polymer and particle surfaces do not establish adequate contact. Therefore, increasing the mixing speed improves efficiency, but still, more intense agitation may mechanically break up the flocs and cause the stretched polymer molecules to readsorb on the same particle surface, preventing further bridging [31]. Consequently, this causes the particles to re-disperse, reducing flocculation efficiency. On an industrial scale, the possible solution could be implementing a multi-stage mixing process, where the high shear rate is applied during the initial phase (to promote dispersion and floc formation), followed by lower shear rates in the later stages, which can help to prevent breakage. A detailed analysis will be performed within the scale-up experiments as a following research step.

Figure 2 graphically presents the influence of the chitosan dosage and the mixing speed on the settling velocity. The results indicated that an increase of the bioflocculant dose is followed by a decrease of the settling time. Chitosan has a positive charge in acidic environments, which allows it to attract negatively charged bacterial cells [32,33]. The settling velocity was 0.03965 mm/s when performing the settling test without flocculant. When different doses of flocculant were used, the range of settling velocities were found to be from 0.03039 to 0.07302 mm/s. Due to disbalance between the amount of cation charge in chitosan and anion charge in bacterial cells, the right quantity of bioflocculant has to be determined to promote floc formation. When the bioflocculant concentration is too high, it does not generate sediment but rather breaks down the precipitate, creating turbidity in the solution. The repulsive attraction occurring among the chitosan positive charges might lead to the deflocculation or restabilization of particles [34]. Particles (flocs) settled faster in experiments with slower agitation rates. Increased mixing speeds have the potential to break apart the flocs, which would affect their settling [30]. However, a reduction in settling velocity occurs when chitosan dosages increase. At greater agitation speeds, the reduction in the settling velocity was more evident. The study results indicate that the chitosan dosage increase between 250 mg/L and 600 mg/L reduced the settling velocity by nearly half for all mixing speed values. This phenomenon is explained by the fact that a higher dosage of chitosan adsorption enhances the positive charge of particles, resulting in a strong electrostatic repulsive force present between the particles and thus preventing their settling [35,36,37]. Surpassing the optimal chitosan concentration (around 220 mg/L) in the case of microalgal–bacterial biomass harvesting from a photobioreactor treating fish processing wastewater resulted in similar observation about particles’ tendency to restabilize due to renewed electrostatic repulsion [30].

Figure 3 illustrates the influence of the chitosan dosage and mixing speed on the flocculated broth’s inhibition zone diameter against *Aspergillus flavus*. The results of the inhibition zone diameter for control tests done with the chitosan solution and cultivation broth (separately) prior to the flocculation experiments were 12.50 ± 0.50 mm and 60.67 ± 0.33 mm, respectively. On the other hand, the results obtained for the flocculated broth indicated greater values of the inhibition zone diameter, implying a synergistic interaction between the cultivation broth and chitosan solution. Similar findings were previously described in the literature [35,38,39]. The lowest speed values combined with the highest chitosan dosages results in the inhibition zone’s maximum value. This pattern is explained by the fact that flocculation effectiveness rises with increasing chitosan dosage; as Figure 1 previously demonstrated, efficiency greater than 90% was obtained for chitosan dosage values over 450 mg/L. The potential toxicity of a high chitosan concentration (up to 672.5 mg/L) to *Bacillus* sp. BioSol021 viability has not been detected. After cultivation, the bacterial concentration was 9.26 log CFU/mL. Following the flocculation experiment, the concentration decreased to 9.1 log CFU/mL when a chitosan concentration of 672.5 mg/L was applied. The bacterial strain demonstrated its viability and maintained effectiveness for its intended application. In addition to microbial cells, the cultivation broth based on complex wastewater effluent contains a range of extracellular compounds and residual nutrients, which interact with chitosan, affecting flocculation efficiency and consequently, antimicrobial activity. A number of these extracellular compounds exhibit antimicrobial activity, contributing to the suppressive activity of the flocculated cultivation broth [9]. The investigation of the mixing speed impact on the inhibition zone diameter revealed an obvious decrease in antimicrobial activity with the mixing speed rise, which is apparent for values higher than 150 rpm. High suspension agitation levels have the potential to break up flocs by intensive mixing, and the extended chitosan molecules may readsorb on the same particle–different place surface, thus preventing bridging [31], as described previously. As a result, the particles’ redispersion occurs, and flocculation efficiency slightly decreases, leading to antimicrobial activity suppression. The antifungal activity of *Bacillus* cells alone remains similar. The decrease of antifungal activity at higher agitation rates (around 9%) can be attributed to the lack of flocculation efficiency of secondary metabolites, such as surfactin, with antifungal activity, which is characteristic of the *Bacillus* sp. BioSol021 strain [24].

The goal of both optimization techniques was to achieve the maximum values of the three responses previously modeled using RSM. The desirability function (DF) was used for optimization of the flocculation operational conditions for the producing microorganism’s biomass harvesting. This optimization method aimed to find optimal experimental conditions for achieving simultaneous maximum values of the three responses modeled utilizing RSM. The DF optimization method was applied to combine multiple desired responses into a single desired response. The selected responses were converted into specific desirability scores in a range from 0 to 1. The overall desirability of the process is determined by taking the geometric mean of each individual desirability function [40]. Initially, the desirability function approach was applied for single responses. The optimal solution (600 mg/L dosage and 100 rpm agitation speed) for maximizing flocculation efficiency alone had a desirability of 0.890. The flocculation efficiency under these conditions was 96.76%. For maximizing the values of the settling velocity, the optimal solution (250 mg/L dosage and 100 rpm mixing speed) had a desirability of 0.596. The settling velocity under these conditions was 0.0556 mm/s. The optimal solution for maximizing the inhibition zone diameter values (591.70 mg/L dosage and 101.81 rpm mixing speed) had a desirability of 1.00 and the inhibition zone diameter under these conditions was 74.4 mm.

According to the established aims and objectives, the process was optimized based on the target values established using predefined criteria—maximizing them, maintaining them within a specific range, and minimizing them [41].

For the simultaneous maximization of all responses, the overall desirability function was 0.491. The optimal solution was obtained at a bioflocculant concentration of 600 mg/L and an agitation speed of 100 rpm. Under these conditions, the efficiency of the flocculation, settling velocity, and inhibition zone diameter were 96.76%, 0.036 mm/s, and 74.61 mm, respectively. The results of the validation experiment showed good agreement with the desirability function predictions, with observed values of 97%, 0.037 mm/s, and 73.82 mm for the efficiency of the flocculation, settling velocity, and inhibition zone diameter, respectively.

The Pareto front results and the desirability function predictions are in good agreement, as illustrated in Figure 4. The trade-off was made in the case of flocculation efficiency, as the lowest value was selected for the lower mixing speed (100 rpm), while the chitosan dosage remained constant at 600 mg/L for all the Pareto optimal front solutions. Nevertheless, the range of optimal solution values for flocculation efficiency is relatively narrow. For the other solution type (higher mixing rates, 200 rpm) the resulting increase of flocculation efficiency (about 1%) resulted in a minor decrease in the other two responses.

Chitosan is recognized as a nontoxic biocompatible and biodegradable polysaccharide with GRAS (Generally Recognized as Safe by the United States Food and Drug Administration) status and proven antimicrobial activity against various pathogens [42]. The antagonistic activity of chitosan against pathogenic fungi is explained through multiple mechanisms of action, including the interactions occurring between the cationic chain and the negatively charged residues of macromolecules placed on the cell surface, causing intracellular content leakage. Additionally, chitosan has an influence on fungal cell wall morphogenesis, directly interfering with key enzymes responsible for fungal growth. Suppression of plant pathogens also happens as a result of plant defense response induction activating the transcription factors after recognition, and chitosan binding by the cell surface receptors [43,44]. Previous studies have confirmed the antagonistic effect of chitosan against *Aspergillus flavus* reaching the maximal inhibition potency of 73.11% [43]. The strong antifungal effect of chitosan is explained by its unique physicochemical properties, and its efficiency level is dependent on the combination of its origin, molecular weight, degree of deacetylation, and pattern of acetylation. Additional parameters are described as fungus-related, taking into account their susceptibility to chitosan. There are two dominant groups, susceptible or sensitive, including most pathogenic fungi, and chitosan-resistant, like nematophagous fungi and insect entomopathogens. Previous studies indicated differences in the cell membrane permeability of those two groups, related to the content of polyunsaturated fatty acids, higher in sensitive species, and saturated palmitic, stearic, and monounsaturated oleic acids, higher in the tolerant ones. Besides species-specific attributes, chitosan suppressive activity is also determined by the developmental stage of fungi. The research showed that conidia treated with chitosan were suppressed within less than 4 min, the conidial germlings within 35–45 min, and the vegetative hyphae within 40 min [45].

Chitosan was the focus of the research addressing the formulation of beneficial bacteria used in plant protection and nutrition, providing improved performance of microbial components and a delivery system for agricultural applications [46]. The combined treatment of *Bacillus velezensis* and chitosan was investigated in greenhouse and field trials treating bread and durum wheat, to reduce *Fusarium* head blight disease severity and deoxynivalenol (DON) accumulation, with the conclusion of the necessity of optimization of the chitosan dosage to achieve the synergistic effect of both components as biocontrol agents [47]. A mixture based on *Bacillus subtilis* HS93 or *Bacillus licheniformis* LS674 and 0.5% chitin significantly contributed to the suppression of development of *Phytophthora* and *Rhizoctonia* pepper root rot, by 62% and 70%, respectively [48].

Biopolymer-based formulations, including chitosan, were previously investigated as effective agents in the encapsulation of beneficial microorganisms, serving as a protective shield against challenging environmental conditions, and resulting in improved viability of cells and overall activity of biopesticides [49]. Additional beneficial effects reflect in the protective function and enhanced stability of metabolites produced by the Bacillus strains, providing improved distribution and permanence in the field, and a longer shelf-life of the final product [50,51].

Creating complex biocontrol products, consisting of microbial cells as active components supplemented by other non-microbial constituents with additional beneficial effects, was defined as an optimal match in terms of not only protecting cells from biotic and abiotic stress, but also boosting their biocontrol activity [52]. Thus, the results of this study offer a promising basis for further exploration of flocculation as an operation with lower techno-economical requirements compared to, e.g., centrifugation, to be included in the downstream processing of microbial biopesticides, being also compliant with the possible application of chitosan as both a flocculation and encapsulation agent during the subsequent product formulation steps, besides its synergistic contribution to biocontrol activity.

## 4. Materials and Methods

### 4.1. Characteristics of Raw Wastewater Substrate

The quality parameters of the FJW used as a basis for microbial biopesticide production, including the pH value (pH meter C860, Consort, Turnhout, Belgium), sugar content [g/L], and dry matter/water content [%, *w*/*v*] were analyzed, and the following values of the investigated parameters were obtained, respectively: 4.92; 2.47^Fructose^, 3.28^Glucose^; and 0.5/99.5.

Sugar substrates (fructose and glucose) in the effluent-based cultivation broth were quantified using HPLC on a Thermo Scientific Dionex UltiMate 3000 system (Thermo Fisher Scientific, Waltham, MA, USA) with a ZORBAX NH2 column (Agilent Technologies, Santa Clara, CA, USA), at the column temperature of 25 °C with a flowrate of 1.2 mL/min using isocratic elution with 75% acetonitrile in the HPLC water as a mobile phase. The detection was performed using a refractive detector (RefractoMax 521, Knauer, Berlin, Germany) with a detector temperature of 25 °C. All cultivation broth samples were centrifuged (12,000× *g*, 10 min, 25 °C, Z 326 K, Hermle LaborTechnik, Wehingen, Germany), and the resulting supernatant samples were filtered through a 0.22 μm membrane prior to autosampler column injection (10 μL).

### 4.2. Biological Treatment of Fruit Juice Industry Wastewater

*Bacillus* sp. BioSol021, originating from the common bean (*Phaseolus vulgaris*) rhizosphere and taxonomically identified as a member of the *Bacillus amyloliquefaciens* operational group (16S rRNA gene sequence accession number ON569805 in the GenBank database), was used as a biopesticide active component in this study [24]. The aflatoxigenic *Aspergillus flavus* PA2D SS, isolated from maize, was used as a test phytopathogen [52].

Cultivation of the producing microorganism (biocontrol strain) was performed using the medium based on wastewater obtained after fruit juice production (FJW), whose initial quality parameters are described above. Prior the cultivation, FJW was supplemented with nutrients replenishing the nitrogen, phosphorus, and microelements essential for microbial growth, as follows: K_2_HPO_4_ was added at the concentration of 0.3 g/L, (NH_4_)_2_SO_4_ was added at the concentration of 3.5 g/L, and MgSO_4_·7H_2_O was added at the concentration of 0.3 g/L. The pH value of the supplemented FJW was initially adjusted using 1 M NaOH to 7.0 ± 0.1 prior to sterilization by autoclaving (121 °C, 2.1 bar, 20 min).

Cultivation of *Bacillus* sp. BioSol021 was carried out in the 16 L lab-scale bioreactor (EDF–15.4_1, A/S Biotehniskais center, Riga, Latvia), whose working volume corresponded to 70% of the total volume. The 100 rpm stirring rate was applied using the Rushton turbine with three impellers, in combination with a 1.5 vvm (volume of sterile air/(volume of the medium·min)) aeration rate for 96 h at the temperature of 28 °C. The fermentation broth, containing the synthesized biomass of the biocontrol agent *Bacillus* sp. BioSol021, was then utilized in downstream processing for the assessment of various flocculation efficiency parameters.

### 4.3. Characteristics of Cultivation Broth

The quality parameters of the *Bacillus* sp. BioSol021 cultivation broth, including the pH value, biomass content [g/L and log CFU/mL], and sugar content [g/L], were determined using the instrumental method (Consort C863, Consort, Turnhout, Belgium), dry weight method, plate count method, and HPLC-based method [24], respectively, and the obtained values of the investigated parameters were 5.66, 3.31, 9.26, and 0.98^Fructose^ and 0.84^Glucose^, respectively.

### 4.4. Coagulation–Flocculation Test

Chitosan (Acros Organics (Geel, Belgium), 90% deacetylated, molecular mass 600,000–800,000 Da) was used both as the coagulant and flocculant in this study. Chitosan was completely dissolved in 0.1 M HCl by continuous stirring on the magnetic stirrer (100 rpm) at room temperature. The chitosan solution (1 mg/mL, kept at 4 °C) was freshly prepared before each flocculation experiment. Flocculation jar tests were performed to investigate and optimize the flocculation process variables (as presented in Table 1) using glass beakers (400 mL) containing the *Bacillus* sp. BioSol021 cultivation broth (250 mL) obtained under the previously described cultivation conditions. Following the addition of the chitosan (dosage varied according to Table 1), the pH value was set to 5.0 ± 0.1 using 0.1 M NaOH/0.1 M HCl. The experiments were started under rapid mixing conditions during the first 5 min using the magnetic stirrer (according to Table 1) for flocculant dispersion, followed by 30 min of slow mixing (50 rpm) to provide conditions for flocculation, while the temperature of 25 °C was maintained in each experiment. Equation (1) was applied to calculate the flocculation efficiency, based on the optical density of the microbial suspension at 600 nm after a settling time of 60 min (UV 1800, Shimadzu, Kyoto, Japan):

(1) $$\%EF=\frac{{OD}_{0}-OD}{{OD}_{0}}\times 100$$

where OD_0_ and OD represent the spectrophotometrically measured optical densities of the microbial suspension samples collected before and after the flocculation, respectively.

### 4.5. Settling Test

Static column settling tests were carried out to determine the settling velocity of the generated flocs, using a column with a height of 20 cm and with an internal diameter of 2.6 cm. The broth samples flocculated using the chitosan were carefully transferred into the column to avoid breakage, followed by the beginning of the settling test when no further disturbances were allowed. The mud line, representing the gradual decline of the solids/liquid interface, was observed and recorded over 60 min in one-minute intervals, as a function of the settling time. The settling velocity was determined using the obtained slope of the linear section of the sedimentation curve [53]. To evaluate the settling velocity of the culture broth, tests were conducted without the addition of flocculants and without agitation.

### 4.6. Antimicrobial Activity

Following the flocculation tests, the antifungal activity of the post-flocculation bacterial biomass–chitosan precipitate against the phytopathogen *Aspergillus flavus* PA2D SS was tested in vitro using the well diffusion method. A homogenized suspension of the fungal pathogen prepared in sterile saline (10^5^ CFU/mL–1 mL) and SMA (Sabouraud Maltose Agar, Himedia Laboratories, Thane, India) media (15 mL) was homogenized and spread in 90 mm Petri plates. Following the medium’s solidification, three 10 mm-diameter wells were created in each plate, followed by the addition of 100 µL of the flocculated sample per well. The inhibition zone diameters were measured after the 96 h incubation period at 26 °C. Control tests were done with the cultivation broth and chitosan solution. The viability of *Bacillus* sp. BioSol021 cells following exposure to the highest applied chitosan concentration of 672.5 mg/L was assessed using the plate count method. The incubation was performed for 48 h at 28 °C.

### 4.7. Modeling and Optimization

The Design-Expert software (v. 8.1, Stat-Ease, Minneapolis, MN, USA) was used for modeling and optimization using the desirability function technique, whilst the MATLAB software (R2015b, MathWorks, Natick, MA, USA) was applied for GA-based optimization.

#### 4.7.1. Experimental Design for Flocculation and RSM Modeling

Polynomial models generated by the response surface methodology (RSM) were applied to establish the link between the variables and responses. Table 1 provides the experimental design data for two independent variables using the central composite design, as well as the selected responses, including the flocculation efficiency, settling velocity, and biocontrol activity in the form of the inhibition zone diameter. The independent variables were the chitosan dosage, in the range of concentrations between 250 and 600 mg/L, and the mixing speed, with values in the range of 100–300 rpm.

The polynomial model, i.e., the second-order polynomial function given by Equation (2), was used to model the responses:

(2) $$Y=b_{0}+b_{1}X_{1}+b_{2}X_{2}+b_{11}X_{1}^{2}+b_{22}X_{2}^{2}+b_{12}X_{1}X_{2}$$

where X_i_ represents the independent variables (X_1_–chitosan dosage (mg/L) and X_2_—mixing speed (rpm)); b_0_ represents the intercept (constant), b_i_ the linear, b_ii_ the quadratic, and b_ij_ the interaction effect of the independent variables; and Y represents the selected responses (flocculation efficiency (%), settling velocity (mm/s), and inhibition zone diameter (mm)).

#### 4.7.2. Optimization by RSM and GA

Optimization of the flocculation parameters was performed using the numerical optimization approach, i.e., using the desirability function (DF) and the genetic algorithm (GA) in combination with the generated response surface models. The RSM approach results in a local solution (from the examined range) to the non-linear model, while, on the other hand, GA results in a set of non-dominated Pareto optimal solutions [27]. The mathematical representation of the studied optimization problem could be presented as follows:

(3) $$\left\{Flocculation\;efficiency\;\left(X_{1},X_{2}\right)\;Settling\;velocity\;\left(X_{1},X_{2}\right)\;Inhibition\;zone\;diameter\;\left(X_{1},X_{2}\right)\right\}\;\left\{250\leq X_{1}\leq 600\frac{mg}{L}100\leq X_{2}\leq 300\;rpm\right\}$$

### 4.8. Statistical Analysis

The selected quadratic polynomial model was statistically analyzed using the analysis of variance (ANOVA) to ensure that each model was adequate to represent the experimental results. The ANOVA procedure is conducted through a series of computations that give information about the degrees of variability considering the selected regression model, and serve as the foundation for significance tests. ANOVA results are usually presented in a table, using the same approach in this study, including the sum of squares (SS), degrees of freedom (DF), and mean square (MS). These parameters are computed for every model and error, while *p*-values are used to perform significance tests.

The statistical significance of the obtained mathematical models and their corresponding coefficients were evaluated using *p*-values, whilst the quality of the models in terms of the fitting of experimental data was judged using pure error, lack-of-fit, and coefficient of determination (R^2^) values. The R^2^ value is the fraction of variability around the dependent variable mean value for which the model could be accounted for, with a range of 0 to 1 (representing the best fit). A chosen second-order polynomial model is not able to precisely fit the obtained experimental values mostly because of measurement errors or correlations between responses and variables that the model cannot represent. This fact results in differences between the expected and observed values, known as residuals. As a result, the residual values appear at the design points [40].

The relevance of the lack-of-fit can be understood when some experimental runs are planned to be replicated in the experimental design (central point replications). The model’s appropriateness is assessed using a statistical test which is based on dividing the residual error SS into two components: the lack-of-fit SS (variation attributable to causes other than measurement error), and the pure error SS (random variation caused predominantly by the measurement error). A low lack-of-fit *p*-value (less than 0.05) indicates that the examined model does not effectively match the experimental data [40,54]. All statistical analyses in this study were done using a 95% threshold of significance.

## 5. Conclusions

The results of the present study indicated that a flocculation efficiency of up to 98.83% could be reached using chitosan as a natural flocculation agent in a concentration of 672.5 mg/L for harvesting *Bacillus* strain biomass cultivated on wastewater from the fruit juice industry. Statistical analyses of the investigated downstream technology revealed that the chitosan concentration primarily influenced the flocculation efficiency and settling velocity, whereas the flocculation rapid agitation speed had a scarce effect on the aforementioned flocculation responses. The obtained experimental results, followed by the applied optimization methodology, support the fact that chitosan, in combination with *Bacillus* sp. BioSol021 biomass, provide synergistic antimicrobial effects against the aflatoxigenic *Aspergillus flavus* tested in this study. The overall desirability function value of the optimized flocculation solution of bacterial broth cultured using the fruit juice wastewater was 0.491, and to achieve this solution, a chitosan dosage of 600 mg/L and a mixing speed of 100 rpm were used. It is indicative that chitosan usage as a flocculation agent represents techno-economically justified downstream processing technology for a growing industry of microbial products for sustainable agriculture based on bacterial biomass. The synergistic effect of microbial and biopolymer components contributes to the overall suppressive activity against the tested fungal phytopathogen, indicating a necessity to investigate a wider range of plant pathogens as possible targets for joint antifungal/antibacterial action in future research endeavors. Future research will aim to investigate the potential for enhancing the circular utilization of the treated liquid residue after the flocculation as a basis for producing a liquid biostimulant derived from the same strain. While the residue retains a small percentage of cells not removed during the flocculation process, it has a reduced nutrient content compared to the original medium. Furthermore, incorporation of the bacterial biomass recovery step based on flocculation, as suggested in this study, in the wider perspective of the biotechnological production chain, should be investigated from the techno-economic perspective, especially taking into account the beneficial circumstances, including the wide application of chitosan as an encapsulating agent, during the formulation of microbial biopesticides.

## Figures and Tables

**Table 1:** The flocculation experimental design—independent variables and responses.

Experiment	Factors—Independent Variables		Responses—Dependent Variables		
	Chitosan Dosage [mg/L]	Rapid Mixing Speed [rpm]	Flocculation Efficiency [%]	Settling Velocity [mm/s]	Inhibition Zone Diameter [mm]
1	250.0	100.0	80.65	0.05333	68.67
2	600.0	100.0	97.18	0.03690	74.00
3	250.0	300.0	80.03	0.04506	66.67
4	600.0	300.0	96.53	0.03229	65.00
5	177.5	200.0	84.02	0.07302	65.00
6	672.5	200.0	98.83	0.04333	71.00
7	425.0	58.60	89.03	0.03556	73.33
8	425.0	341.4	87.86	0.03778	66.67
9	425.0	200.0	91.44	0.03385	66.00
10	425.0	200.0	90.93	0.03039	65.33

**Table 2:** Analysis of variance (ANOVA) for the response flocculation efficiency.

Source	DF	SS	MS	F-Value	*p*-Value
Model	5	379.9947	75.9989	13.8272	0.0124
Residual	4	21.9853	5.4963		
Lack-of-fit	3	21.8553	7.2851	56.0176	0.0978
Pure error	1	0.1301	0.1301		
Total	9	401.9800			
	R^2^		Adj. R^2^	Adeq. Precision	
	0.9453		0.8769	10.5085	

DF—degrees of freedom, SS—sum of squares, MS—mean square.

**Table 3:** Regression equation coefficients for the response flocculation efficiency.

Effects	Coefficient		*p*-Value
	Actual	Coded	
Intercept			
*b*_0_	67.4120	91.1851	<0.0001
Linear			
*b*_1_	0.0446	6.7466	0.0012
*b*_2_	0.0647	−0.3656	0.6820
Quadratic			
*b*_11_	−7.00 × 10^−6^	−0.2142	0.8546
*b*_22_	−0.0002	−1.7049	0.1950
Interaction			
*b*_12_	−4.29 × 10^−7^	−0.0075	0.9952

**Table 4:** Analysis of variance (ANOVA) for the response settling velocity.

Source	DF	SS	MS	F-Value	*p*-Value
Model	5	0.0014	0.0003	9.1225	0.0262
Residual	4	0.0001	2.99 × 10^−5^		
Lack-of-fit	3	0.0001	3.78 × 10^−5^	6.3129	0.2827
Pure error	1	5.99 × 10^−6^	5.99 × 10^−6^		
Total	9	0.0015			
	R^2^		Adj. R^2^	Adeq. Precision	
	0.9194		0.8186	8.4735	

DF—degrees of freedom, SS—sum of squares, MS—mean square.

**Table 5:** Regression equation coefficients for the response settling velocity.

Effects	Coefficient		*p*-Value
	Actual	Coded	
Intercept			
*b*_0_	0.1328	0.0321	<0.0001
Linear			
*b*_1_	−0.0004	−0.0089	0.0100
*b*_2_	−0.0001	−0.0012	0.5627
Quadratic			
*b*_11_	3.80 × 10^−7^	0.0116	0.0104
*b*_22_	8.91 × 10^−8^	0.0009	0.7451
Interaction			
*b*_12_	523 × 10^−8^	0.0009	0.7547

**Table 6:** Analysis of variance (ANOVA) for the response inhibition zone diameter.

Source	DF	SS	MS	F-Value		*p*-Value
Model	5	102.4538	20.4908	21.6161		0.0054
Residual	4	3.7918	0.9479			
Lack-of-fit	3	3.5673	1.1891	6.3129		0.3067
Pure error	1	0.2245	0.2245			
Total	9	106.2456				
	R^2^		Adj. R^2^		Adeq. Precision	
	0.9643		0.9197		11.8916	

DF—degrees of freedom, SS—sum of squares, MS—mean square.

**Table 7:** Regression equation coefficients for the response inhibition zone diameter.

Effects	Coefficient		*p*-Value
	Actual	Coded	
Intercept			
*b*_0_	73.1125	65.6650	<0.0001
Linear			
*b*_1_	−0.0008	1.5181	0.0116
*b*_2_	−0.0656	−2.5525	0.0018
Quadratic			
*b*_11_	3.47 × 10^5^	1.0635	0.0797
*b*_22_	0.0002	2.0642	0.0106
Interaction			
*b*_12_	0.0002	−1.7500	0.0229

## Data Availability

The original contributions presented in this study are included in the article. Further inquiries can be directed to the corresponding author(s).
